# Molecular Characterization of New FBXL4 Mutations in Patients With mtDNA Depletion Syndrome

**DOI:** 10.3389/fgene.2019.01300

**Published:** 2020-01-08

**Authors:** Sonia Emperador, Nuria Garrido-Pérez, Javier Amezcua-Gil, Paula Gaudó, Julio Alberto Andrés-Sanz, Delia Yubero, Ana Fernández-Marmiesse, Maria M. O’Callaghan, Juan D. Ortigoza-Escobar, Marti Iriondo, Eduardo Ruiz-Pesini, Angels García-Cazorla, Mercedes Gil-Campos, Rafael Artuch, Julio Montoya, María Pilar Bayona-Bafaluy

**Affiliations:** ^1^Departamento de Bioquímica, Biología Molecular y Celular, Universidad de Zaragoza, Zaragoza, Spain; ^2^Instituto de Investigación Sanitaria de Aragón (IIS-Aragón), Zaragoza, Spain; ^3^Fundación ARAID, Universidad de Zaragoza, Zaragoza, Spain; ^4^Clinical Biochemistry, Genetics, Pediatric Neurology and Neonatalogy Departments, Institut de Recerca Sant Joan de Déu, Barcelona, Spain; ^5^Centro de Investigaciones Biomédicas en Red de Enfermedades Raras (CIBERER), Madrid, Spain; ^6^Genomes&Disease Group, Molecular Medicine and Chronic Diseases Research Centre (CiMUS), Santiago de Compostela University—IDIS, Santiago de Compostela, Spain; ^7^Metabolism Unit, Reina Sofia University Clinical Hospital, Institute Maimónides of Biomedicine Investigation of Córdoba (IMIBIC), University of Córdoba, Córdoba, Spain; ^8^CIBEROBN (Physiopathology of Obesity and Nutrition CB12/03/30038), Madrid, Spain

**Keywords:** mitochondrial disease, encephalomyopathic mtDNA depletion syndrome 13, F-box leucine-rich repeat protein 4, mitochondrial DNA, mtDNA depletion, mtDNA transcription, oxidative phosphorylation

## Abstract

Encephalomyopathic mitochondrial DNA (mtDNA) depletion syndrome 13 (MTDPS13) is a rare genetic disorder caused by defects in F-box leucine-rich repeat protein 4 (FBXL4). Although FBXL4 is essential for the bioenergetic homeostasis of the cell, the precise role of the protein remains unknown. In this study, we report two cases of unrelated patients presenting in the neonatal period with hyperlactacidemia and generalized hypotonia. Severe mtDNA depletion was detected in muscle biopsy in both patients. Genetic analysis showed one patient as having in compound heterozygosis a splice site variant c.858+5G>C and a missense variant c.1510T>C (p.Cys504Arg) in *FBXL4*. The second patient harbored a frameshift novel variant c.851delC (p.Pro284LeufsTer7) in homozygosis. To validate the pathogenicity of these variants, molecular and biochemical analyses were performed using skin-derived fibroblasts. We observed that the mtDNA depletion was less severe in fibroblasts than in muscle. Interestingly, the cells harboring a nonsense variant in homozygosis showed normal mtDNA copy number. Both patient fibroblasts, however, demonstrated reduced mitochondrial transcript quantity leading to diminished steady state levels of respiratory complex subunits, decreased respiratory complex IV (CIV) activity, and finally, low mitochondrial ATP levels. Both patients also revealed citrate synthase deficiency. Genetic complementation assays established that the deficient phenotype was rescued by the canonical version of *FBXL4*, confirming the pathological nature of the variants. Further analysis of fibroblasts allowed to establish that increased mitochondrial mass, mitochondrial fragmentation, and augmented autophagy are associated with FBXL4 deficiency in cells, but are probably secondary to a primary metabolic defect affecting oxidative phosphorylation.

## Introduction

A dysfunction in the maintenance of the mitochondrial DNA (mtDNA) leads to the reduction of mtDNA copy number and/or the accumulation of defects in mtDNA. mtDNA depletion syndromes (MDSs) are a group of mitochondrial disorders characterized by a severe loss of mtDNA copy number. MDSs are autosomal recessive disorders, genetically heterogeneous, and clinically presented in encephalomyopathic, hepatocerebral or myopathic forms (Suomalainen and Isohanni, 2010; Viscomi and Zeviani, 2017).

The human mtDNA contains genetic coding information for 13 proteins, which are core constituents of the mitochondrial respiratory complexes I, III, and IV (CI, CIII, and CIV) and the F_1_F_0_-ATPsynthase [complex V (CV)]. The respiratory complexes are embedded in the inner mitochondrial membrane and function together with the tricarboxylic acid (TCA) cycle in the matrix. The TCA cycle, together with the beta oxidation of fatty acids, is pivotal for generation of NADH and FADH_2_ to be oxidized by the respiratory chain. The electron flux along the chain creates an electrochemical gradient that powers the synthesis of most cellular ATP by CV [oxidative phosphorylation (OxPhos)]. mtDNA depletion therefore causes a combined respiratory chain deficiency and deficiency of oxidative ATP-synthesis.

The study of pathogenic variants in patients with defects in mtDNA maintenance has shown that this process depends on a number of nuclear gene-encoded proteins that function in mtDNA synthesis, either participating in mtDNA replication or in the maintenance of balanced nucleotide pools, which constitute the necessary building blocks (Suomalainen and Isohanni, 2010). Qualitative defects in mtDNA (multiple mtDNA deletions) can in addition be caused by defects in mitochondrial division and fusion processes that influence mtDNA segregation (El-Hattab et al., 2017a; Viscomi and Zeviani, 2017).

Defects in F-box leucine-rich repeat 4 (FBXL4) protein, whose molecular function has yet to be determined, cause an encephalomyopathic type of MDS (MTDPS13; OMIM # 615471). MTDPS13 commonly presents with hypotonia, psychomotor delay, failure to thrive, feeding difficulties, growth failure, and lactic acidosis, among other less common manifestations (El-Hattab et al., 2017a). The age of onset ranges from birth to 2 years (mean 4 months). More than a third of affected children die during childhood and long-term survivors develop severe psychomotor retardation. In skeletal muscle tissue commonly appear cytochrome oxidase (COX)-deficient fibers, decreased activities of all respiratory chain enzymes, particularly CI and CIV, and mtDNA depletion (El-Hattab et al., 2017a). The study of cells derived from affected patients demonstrated that FBXL4 is a mitochondrial protein controlling bioenergetic homeostasis and mtDNA maintenance (Bonnen et al., 2013; Gai et al., 2013). However, the molecular role of FBXL4 in mtDNA maintenance remains unclear.

Here we report the identification of novel *FBXL4* mutations in two independent patients and supported the causal role of those mutations. The study of patient derived fibroblasts provided some clues to understand the molecular function of the protein.

## Materials and Methods

### Cell Culture and Cell Staining With Fluorescent Dyes

S1, S2, and C1 primary skin-derived fibroblasts were obtained from Subject 1, Subject 2, and a 1 month-old control child, respectively. C refers to mix of three primary fibroblasts from 1 month, 3 years and 38 years old controls, respectively. Cells were cultured at 37°C under a 5% CO_2_ atmosphere in high-glucose DMEM medium (Gibco-ThermoFisher Scientific) with 10% fetal bovine serum (FBS; Gibco-ThermoFisher Scientific).

Cell staining was performed in six well plates. Logarithmically growing cells were incubated with FBS free DMEM for 30 min at 37°C and then stained for 30 min at 37°C in the dark with 200 nM of either MitoTracker™ Green (Invitrogen) or MitoTracker™ Red CMXRos (Invitrogen) in the culture medium. For flow cytometry, immediately after staining, cells were collected by trypsinization and 10,000 particles were analyzed with a Beckman Coulter CITOMICS FC 500 Flow Cytometer. For fluorescent microscopy, cells grown and stained over cover-slides were fixed following a standard protocol and images were obtained with a ZEISS HAL100 microscope.

### Biochemical Analysis

Blood lactate values were determined by automated spectrophotometry. Plasma amino acids and urine organic acids were analyzed by ion exchange chromatography with ninhydrin detection derivatives and gas chromatography/mass spectrometry, respectively.

### Genomic Analysis

Nuclear DNA (nDNA) was assessed by next generation sequencing (NGS) using customized gene panels as previously reported (Yubero et al., 2016; Fernandez-Marmiesse et al., 2019), in a NextSeq500 sequencer (Illumina).

### Alignment of FBXL4 Reference Sequences

Chordate FBXL4 reference sequences (243) were obtained from GenBank (http://www.ncbi.nlm.nih.gov/genbank/) (accessed July 22^nd^, 2019), and aligned with Clustal Omega (https://www.ebi.ac.uk/Tools/msa/clustalo/).

### Analysis of *FBXL4* Transcripts and Genetic Complementation

The *FBXL4* cDNA (corresponding to RefSeq NM_012160.4; NP_036292.2) was amplified from retrotranscribed total RNA of control and patient fibroblasts, as in (Emperador et al., 2014), using the specific primers: Fw: GATATCGCCACCATGTCACCGGTCTTTCC and Rv: GATATCTCACTGAGTAAAGCTC. After cloning with the TOPO™ PCR Cloning system (Invitrogen), six to eight bacterial clones per cell line were isolated and their plasmids sequenced.

For genetic complementation, a sequence checked clone, obtained from control fibroblasts, was transferred to the lentiviral expression vector pWPXLd-ires-Neo^R^, that is a modified version of pWPXLd (Tronolab, Addgene #12258). Lentiviral particles were generated as in (Perales-Clemente et al., 2008) and fibroblasts were transduced with lentiviral particles in 100 mm dishes by adding 10 μl of medium with viral particles. Transduced cells were isolated by 10 days selection in the presence of 400 μg/ml geneticin (Invitrogen-ThermoFisher Scientific).

### Real Time Quantitative Polymerase Chain Reaction Experiments

mtDNA copy number was quantitated by quantitative polymerase chain reaction (qPCR) as previously described (Andreu et al., 2009), using a StepOne™ Real-Time PCR System (Applied Biosystems™). The mitochondrial probe, labeled with a FAM fluorophore, was targeted to the *MT-RNR1* gene (TGC CAG CCA CCG CG) and the nuclear probe, labeled with a VIC was targeted to the *RNAsa P* gene.

To assess mitochondrial mRNA levels, total RNA was isolated from exponentially growing cells using a NucleoSpin^®^ RNA II kit (Macherey-Nagel). Total RNA (1 μg) was reversed-transcribed (RT) with the Transcriptor First Strand cDNA Synthesis Kit (Roche). The levels of *MT-ND1*, *MT-ND6*, *MT-CYB*, *MT-CO1*, and *MT-ATP6* were determined by RT-qPCR using the One-Step Real-Time system (Applied Biosytems). The expression levels were normalized using the 18S ribosomal RNA. The ΔΔCt method was used to calculate fold expression. StepOne software version 2.0 (Applied Biosystems) was used for data analysis. To quantify *FBXL4* transcripts qPCR was carried out in a LightCycler 2.0 system (Roche), using the specific primers: qFw: TGAGATGTGTCCAAATCTACAGG and qRv: GCTGAGCAGTGCTGTTTGC.

### SDS-PAGE and Western Blot Analysis

For Western blotting (WB), 20 μg of either total cellular protein extracted in RIPA buffer (MILLIPORE), or total cell homogenate treated by freeze-thawing (4X) (for LC3B WB) was separated in 12.5% acrylamide/bis-acrylamide SDS/PAGE, electroblotted onto PVDF filter, and sequentially probed with specific antibodies: anti-FBXL4 (Sigma, #SAB2701256), anti-OXPHOS cocktail (Abcam, #ab110411), anti-SDHA (Thermo Fisher Scientific, #459200), anti-Actin (Sigma, #A 2066), anti-CS (Sigma, # SAB2702186), anti-TOMM20 (SantaCruz biotechnology, Inc., #sc-11415), and anti-LC3B (Sigma, #L7543). Luminescence images were acquired using Amersham Imager 600 (GE Healthcare Life Sciences) and quantitative data were obtained with ImageQuant™ TL 8.1 analysis software.

### Complex IV Levels and Complex IV and Citrate Synthase Specific Activities

When Microplate Assays were indicated, complex IV (CIV) activity and levels were measured using the CIV Specific Activity Microplate Assay Kit (Mitosciences, Abcam^®^), according to the manufacturer's instructions, and CS was measured in 96 well plates, using freeze-thawing treated total cell homogenate and a standard protocol (Kirby et al., 2007). Microplate assays were performed in a NovoStar MBG Labtech microplate instrument. Otherwise, CIV and CS activities were measured in an UNICAM UV 500 spectrophotometer using digitonin (Sigma) solubilized cell samples as described previously (Kirby et al., 2007). Activity data were normalized for total protein.

### ATP Measurements

ATP levels were measured using the ATP bioluminescence assay kit CellTiter-Glo^®^ Luminescent Cell Viability Assay (Promega), according to the manufacturer's instructions. Values were normalized using the CellTiter-Blue^®^ Cell Viability Assay (Promega) according to the manufacturer's instructions. Samples were measured using a NovoStar MBG Labtech microplate instrument.

### Statistical Analysis

The statistical package StatView 6.0 was used to perform the statistical analysis. Data are expressed as mean ± SD (standard deviation). The non-parametric Mann-Whitney test was used to evaluate the statistical significance between experimental groups. *P*-values lower than 0.05 were considered statistically significant. All samples were measured at least in biological triplicates.

## Results

### Subject Description

**S1:** This female child, born to nonconsanguineous parents of European ancestry, was delivered at 38 weeks gestation with a very low weight for gestational age (2.650 kg, < 1^st^ percentile). Fetal ultrasounds also reported a single umbilical artery and megacisterna magna. In the first days of life, mild hypotonia and nystagmus triggered by Moro reflex were observed. Blood lactate was repeatedly increased (4.8 to 11.3 mmol/L; reference values (RV) < 2.2) along with alanine. Brain magnetic resonance image (MRI) revealed mild cerebellar hypoplasia and probable bilateral simplified temporal and frontal gyration pattern. These results led to the study of mitochondrial disease. At 2 months of age, she had frequent visits to the Emergency Department due to intercurrent respiratory processes. Psychomotor development was delayed with poor eye contact and hypotonia. At 6 months of age, she presented with infantile spasms (West syndrome) that responded to treatment with vigabatrin and prednisolone. Due to metabolic acidosis, treatment with bicarbonate and L-carnitine was initiated. In addition, she had gastroesophageal reflux with frequent vomiting. She presented with progressive dysphagia with poor control of respiratory secretions, convergent strabismus as well as brain MRI lesions compatible with Leigh syndrome. At 10 months, there was an episode of aspiration with marked deterioration in her general condition, generalized hypotonia and seizures. A worsening of the brainstem lesions was observed on brain MRI. The symptoms were progressive with encephalopathy, metabolic acidosis, and death.

**S2:** This male child, born to consanguineous parents of Moroccan ancestry, was delivered by caesarean section due to arrest of dilation at 38.4 weeks gestation with a low weight for gestational age (2.650 kg, 8^th^ percentile), short length for gestational age (47 cm, 5^th^ percentile), and normal head circumference (34 cm, 43^rd^ percentile). On the third day of life, he showed an acute neurological and respiratory worsening with cyanosis and hypotonia. The patient was intubated and ventilated. Physical examination showed clinical signs of poor peripheral perfusion and absence of responses to stimuli due to sedoanalgesia. On admission, he presented with metabolic acidosis (pH 7.13, pCO_2_ 22.2 mmHg on mechanical ventilation, stHCO_3_ 8.9 mmol/L, EB –20.6 mmol/L), hyperamonemia (224 µmol/L: RV <70), and hyperlactacidemia (25 mmol/L: RV < 2.2). Plasma amino acids exhibited increased alanine (1385 µmol/L: RV 190–337), glycine (658 µmol/L: RV 180–291), glutamine (971 µmol/L: RV 420–750), and lysine (705 µmol/L: RV 67–202). Organics acids revealed a massive accumulation of lactic, 3-hydroxybutyric, acetoacetic, and 2-hydroxybutyric acids, leading to investigate mitochondrial diseases. No analytical signs of infection were detected. Transfontanellar ultrasound showed extensive hyperechogeneity that seemed to correspond to retro cerebellar hemorrhage and enlarged magna cisterna. No brain MRI or lumbar puncture was performed. Echocardiography showed pulmonary hypertension.

Different treatment approaches were initiated to correct the metabolic abnormalities: protein restriction, high energy intake (100–120 cal/kg/day), L-carnitine, arginine, and vitamins (biotin, hydroxycobalamin, pyridoxine, riboflavin, and thiamine). An intravenous insulin and dopamine (maximum dose 5 µg/kg/min) pump and antibiotics (ampicillin and gentamicin) were also started. He received several doses of intravenous bicarbonate and three doses of adrenaline for severe bradycardia. At 24 hours of admission, there was a limitation of the therapeutic effort due to the refractoriness of lactic acidosis. The child died at the age of 4 days.

Although some dysmorphisms have been reported in FBXL4 patients, microcephaly, cataracts, malformed ears, or other dysmorphic facial features were not found in S1 or S2 patients. However, S2 showed distal hypospadias and bilateral cryptorchidism.

### Biallelic Mutations in FBXL4 Are Present in the Probands

**Figure 1:**
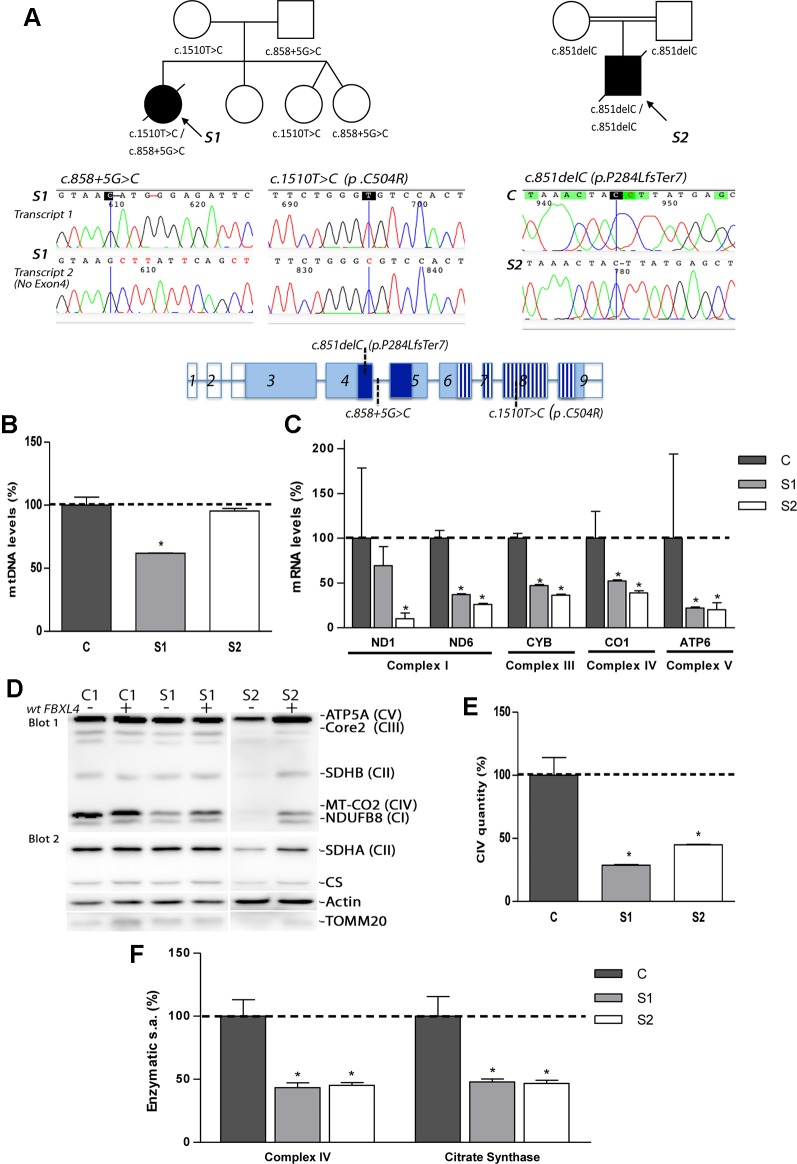
Genetic, molecular, and biochemical characterization of the patients. **(A)** Pedigrees of S1 and S2 with genotypes indicated under each symbol (black symbols designate affected subjects); sequencing electropherograms corresponding to the two different FBXL4-derived transcripts found in S1 and to the unique transcript found in S2; and FBXL4 gene structure (reference sequence NM_012160.4). In gene structure, empty boxes: non-coding exons; dark blue boxes: F-Box domain; striped boxes: leucine-rich repeats domain (LRRs). **(B)** Quantification of mtDNA copy number. The bars represent percentage of mtDNA normalized to nDNA relative to the mean value of controls levels (C, dotted line, 100%). *: Significant mtDNA copy-number reduction, p < 0.05, compared with C cells. **(C)** Mitochondrial transcript levels. For each transcript, the bars represents mean values, in percentage, relative to the mean value of control fibroblasts (C, dotted line, 100%). *: significant mtDNA reduction, p < 0.05, compared with C cells. **(D)** Steady-state levels of mitochondrial respiratory chain subunits. WB-immunodetection of SDS-PAGE separated total cellular protein isolated from patient S1, S2 and C fibroblasts (−), and those transduced with wt-FBXL4 expressing construct (+). An OXPHOS cocktail of antibodies was used in the upper membrane (blot 1) and the indicated antibodies were sequentially used in the lower membrane (blot 2). **(E)** Complex IV quantity (Microplate Assay). The bars represent the mean value of S1 and S2, in percentage, compared to that of controls fibroblasts (dotted line, 100%). *: p < 0.05 (vs. C cells). **(F)** Complex IV (CIV) and CS specific activities (s.a.) (Microplate Assay). The bars represent enzymatic activity of CIV and CS normalized for total cellular protein and compared to the mean value of controls fibroblasts in percentage (dotted line, 100%). *: p < 0.05 (vs. C cells).

Exome sequencing analysis of S1 and S2 detected novel mutations in *FBXL4* (**Figure 1A**). S1 was found to be compound heterozygous for the split site variant c.858+5G>C and the missense variant c.1510T>C (p.Cys504Arg). The first variant was detected in the father and the second in the mother. S2 was found to be homozygous for the frameshift variant c.851delC (p.Pro284LeufsTer7), that was detected in both parents in heterozygosis. The variants were confirmed by Sanger sequencing.

Patient RNA-retrotranscription and cDNA cloning revealed two classes of *FBXL4* transcripts in S1 (**Figure 1A**). One transcript harbored the missense variant c.1510T>C (p.Cys504Arg) and a second one lacked exon 4, which can be compatible with a splicing defect. The analysis of S2 detected only one type of transcript harboring the frameshift variant c.851delC (p.P284LfsTer7) (**Figure 1A**).

None of these variants were present in the ExAC browser (accessed July 2019). However, the variant producing an unexpected transcript in S1 has been previously published in heterozygosis in a patient with MTDPS13 (rs1257765682) (Pronicka et al., 2016). The S1 missense variant affects a position conserved in 243 out of 243 reference sequences and is considered pathogenic by several prediction software packages (MutationTaster, PMut and PolyPhen-2). Eighteen months after the death of S1, the parents had a healthy girl that was not a carrier of either of the two mutations. Two years later, the parents had a new twin pregnancy. The amniocentesis of both fetus revealed that they were only carriers of the c.858+5G>C variant inherited from the father. At 2-years of age, both of them are healthy.

### Defective OxPhos System Biogenesis Is Associated With the Novel *FBXL4* Mutations

Analysis of mtDNA copy number revealed severe mtDNA depletion in muscle biopsies of the patients (85% in S1 and 93% in S2). In cultured skin fibroblasts, milder mtDNA depletion was detected in S1 (38%) whereas normal levels of mtDNA were observed in S2 (**Figure 1B**). The levels of five mitochondrial transcripts (transcripts of *MT-ND1* and *MT-ND6* subunits from CI; *MT-CYB* subunit from CIII, *MT-CO1* subunit from CIV and *MT-ATP6* subunit from CV) were consistently reduced in S1 and S2 compared with control fibroblasts (**Figure 1C**). Notably, S2, with normal mtDNA copy number, showed the highest reduction of the five transcripts measured.

The steady-state levels of subunits from respiratory chain complexes were also found decreased in fibroblasts from S1, and, to a greater extent, in fibroblasts from S2 (**Figure 1D**). The levels of the nDNA-encoded ATP5A subunit from CV, however, remained unchanged in S1, or were mildly decreased in S2, excluding a global problem in the mitochondrial protein content. The expression of two subunits (SDHA and SDHB) of the nuclear encoded complex II, the citrate synthase (CS) of the TCA cycle, and the translocase of the outer mitochondrial membrane (TOMM20) were partially decreased in S2 (**Figure 1D**). Fully assembled CIV levels were clearly lower in patient fibroblasts relative to controls (**Figure 1E**). Enzymatic measurements provided evidenced of a severe CIV dysfunction (**Figure 1F**). Noteworthy, the CS activity in fibroblasts from S1 and S2 was also significantly diminished (**Figure 1F**).

These results indicated that a decreased expression of mtDNA-encoded genes affects the correct biogenesis and function of the respiratory chain, with the TCA-cycle enzyme CS also affected.

### Delivery of Wild-Type *FBXL4* Corrects the OxPhos Dysfunction

**Figure 2:**
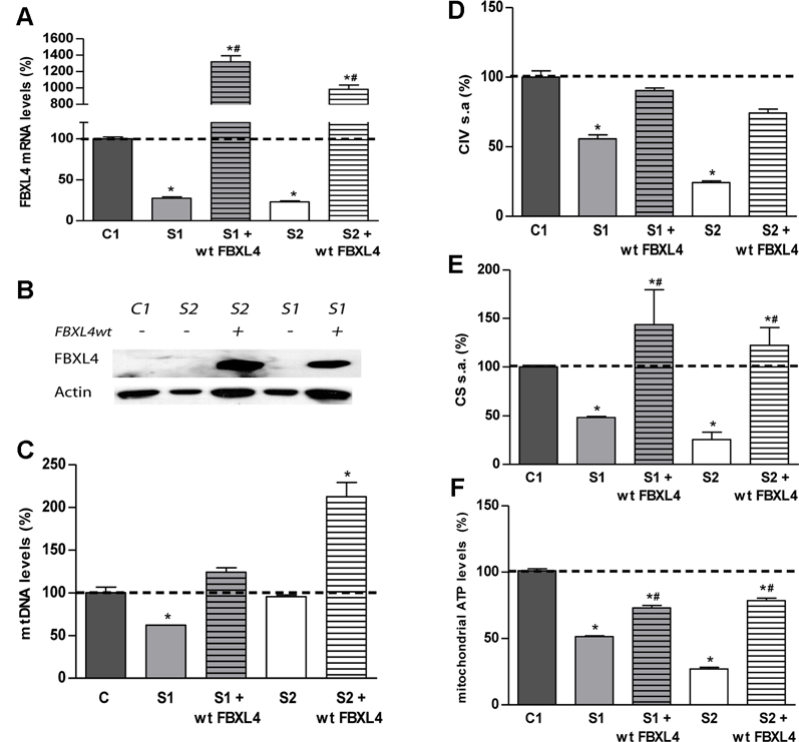
FBXL4 complementation assays. **(A)** *FBXL4* expression levels. Bars represent *FBXL4* mRNA levels of patient fibroblasts compared to the mean value of an age-matched control in percentage (C1 dotted line, 100%). *: p < 0.05 (vs. C1 cells); #: p < 0.05 (vs. non-transduced cells). **(B)** FBXL4 protein levels. WB-immunodetection with anti-FBXL4 antibody of total cellular proteins isolated from S1, S2, and C1 fibroblasts, and those transduced with wt-*FBXL4* expressing construct. **(C)** Quantification of mtDNA copy number of S1, S2, and C1 fibroblasts and those transduced with the wt-*FBXL4* expressing construct. Bars represent the mean value of mtDNA normalized to nDNA, in percentage, relative to the mean value of controls levels (C, dotted line, 100%). *: p < 0.05 (vs. C cells); #: p < 0.05 (vs. non-transduced cells). **(D)** Complex IV specific activity of S1, S2, and C1 fibroblasts and of those transduced with the wt-*FBXL4* expressing construct. Bars represent the mean value relative to that of control fibroblasts in percentage (C1, dotted line, 100%). *: p < 0.05 (vs. C1 cells). **(E)** CS specific activity of S1, S2, and C fibroblasts and those transduced with the wt-*FBXL4* expressing construct. Bars represent the mean value relative to that of control fibroblasts, in percentage (C1, dotted line, 100%). *: p < 0.05 (vs. C1 cells); #: p < 0.05 (vs. non-transduced cells). **(F)** Mitochondrial ATP levels. Bars represent the mean values in S1, S2 and C1, S1 and S2 transduced with the wt-*FBXL4* expressing construct, relative to that of control cells in percentage (C1, dotted line, 100%). *: p < 0.05 (vs. C1 cells); #: p < 0.05 (vs. non-transduced cells).

In order to confirm the pathogenicity of the FBXL4 mutations, we performed genetic complementation studies. Stably transduced patient cells showed a robust expression of wild-type *FBXL4* (wt-*FBXL4*) at transcript and protein levels (**Figures 2A, B**). The quantification of *FBXL4* transcripts by qRT-PCR revealed decreased steady-state levels of *FBXL4* mRNA in S1 and S2 by approximately 80% when compared with an age matched control cell line, C1. These levels were increased significantly in the over-expressing cell lines (**Figure 2A**). The WB-inmunodetection assay failed to detect the FBXL4 protein in the total protein lysate of non-transduced cells (**Figure 2B**).

Delivery of the wt-*FBXL4* gene increased significantly the amount of mtDNA in both patient cell lines (**Figure 2C**). As a result, the mtDNA copy number deficiency in S1 cells was corrected (compared to C), whereas in S2 cells the mtDNA levels increased up to 200% of the levels of controls. In both S1 and S2 cells over-expressing wt-*FBXL4*, the steady state levels of respiratory complex subunits were fully rescued (**Figure 1D**), the CIV specific activity was increased to control values (**Figure 2D**), and the CS activity and lastly the mitochondrial ATP levels were also increased significantly (**Figures 2E, F**).

These results confirmed the causal role of the mutations identified in FBXL4 in the metabolic dysfunction.

### Mitochondrial Mass, Mitochondrial Fragmentation, and Autophagosomes Are Increased in FBXL4 Deficiency

**Figure 3:**
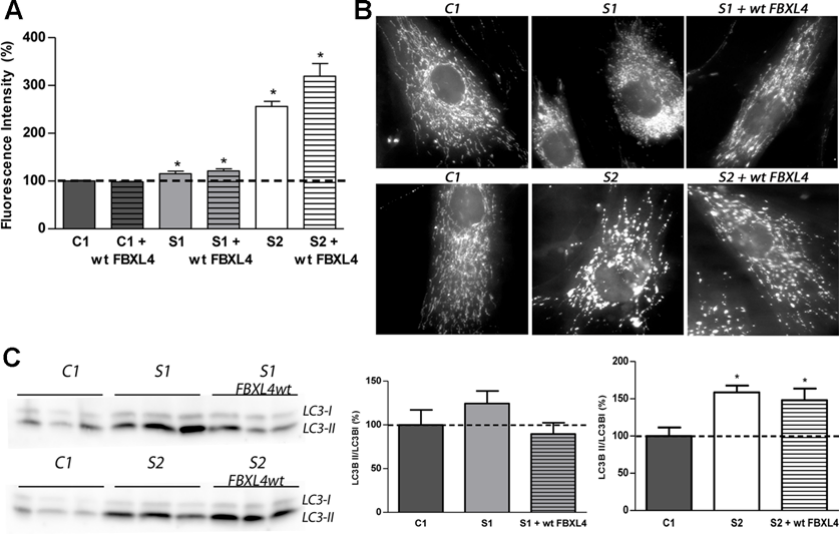
Mitochondrial network shape and size, and autophagy detection. **(A)** Quantification of mitochondrial mass. Bars represent the mean fluorescence values relative to the mean value of age-matched control fibroblasts in percentage (C1, dotted line, 100%). *: p < 0.05 (vs. C1 cells). **(B)** Mitochondrial networks of control and patients fibroblasts. Fluorescence microscopy representative images of cells obtained from control and patient fibroblasts and of those transduced with the wt-*FBXL4* expressing construct, as indicated. **(C)** Quantification of autophagy marker LC3-II. WB-immunodetection of the LC3B isoforms (LC3B-I and LC3B-II) of total cell homogenates, and ratio LC3B-II/LC3B-I obtained by quantification of the respective WB-band intensities. The bars represent the mean value, in percentage, compared to that of control fibroblasts (dotted line, 100%). *: p < 0.05 (vs. C1 cells).

Next, we investigated the mitochondrial mass in cultured patient fibroblasts to determine whether it is reduced as could be suggested by the low CS activity. Assessment of mitochondrial content by staining with the cationic lipophilic dye MitoTracker Green failed to show differences between C1 and wt-*FBXL4* overexpressing C1 cells (**Figure 3A**). However, a small but significant increase in mitochondrial content (15–20%) was detected in S1 and complemented S1 cell lines compared to C1, and a remarkable increase (250% of C1) was detected in S2 fibroblasts and in the S2 cell line overexpressing wt-*FBXL4*.

The mitochondrial network morphology was next examined. As shown in the fluorescence microscopy images (**Figure 3B**), the fluorescent pattern obtained with the mitochondrion-specific dye MitoTracker was mainly tubular in C1 cells. On the contrary, S1 and S2 cell lines showed fragmentation of the mitochondrial network. Genetically complemented S1 cells recovered partially the tubular appearance of the mitochondrial network observed in C1 but interestingly, genetically complemented S2 cells maintained a fragmented mitochondrial network.

The autophagosome marker MAP1 light chain 3B (LC3B) can be found as LC3B-I, mainly cytosolic, or as LC3B-II, which is covalently attached to phosphatidylethanolamine and coats the surface of autophagosomes. Western blotting and immunodetection were used to analyze LC3B-I to LC3B-II conversion and estimate the abundance of autophagy-related structures (Klionsky et al., 2008). The ratio LC3B-II/LC3B-I was found augmented in S1 and S2 cells (**Figure 3C**). Albeit in genetically complemented S1 cells, the ratio was reduced to the control levels; in genetically complemented S2 cells, however, the significantly high levels of autophagy marker LC3-II related to its cytosolic isoform LC3-I persisted.

These results suggested that mitochondrial fragmentation and an increase of autophagosomes are associated with FBXL4 mutations in cultured fibroblasts. Remarkably, the genetically complemented S2 cell line failed to recover normal mitochondrial size or shape and normal autophagy levels, suggesting that these are secondary adaptations to the metabolic dysfunction promoted by FBXL4 deficiency.

## Discussion

This work presents two patients with severe encephalopathy and severe mtDNA depletion in muscle associated with novel variants in *FBXL4*; S1 harboring two variants in compound heterozygosis c.[858+5G>C];[1510T>C] and S2 harboring one variant in homozygosis c.[851delC];[851delC]. The pathogenicity of the variants was confirmed by genetic complementation assays in skin-derived fibroblasts. Genetic complementation studies with this gene have only been performed in three subjects previously (Bonnen et al., 2013; Gai et al., 2013).

A review of genotype–phenotype correlation in 87 affected individuals with pathological variants in *FBXL4* indicated that genotypes with missense variants are frequently associated with longer survival (El-Hattab et al., 2017b). Thus, the missense variant encountered in S1 could be associated with preservation of some residual protein function and therefore with a milder phenotype. However, both patients presented with very severe clinical phenotype associated with very early onset and short survival (S1, 10 months, and S2, 4 days), indicating that any possible residual function of the S1 missense variant was not sufficient to maintain *in vivo* protein function.

The finding of severe mtDNA depletion in skeletal muscle biopsies (85% in S1 and 93% in S2) are in line with published data indicating an essential role of FBXL4 in the maintenance of mtDNA. Interestingly, important differences were observed in the mtDNA content of cultured fibroblasts. S1 cells presented with quantitative mtDNA copy number reduction (38%), whereas S2 cells had normal mtDNA copy number. Remarkably, genetic complementation increased the mtDNA levels in at least 50%, still indicating an involvement of FBXL4 in mitochondrial mtDNA copy number maintenance. Despite having normal mtDNA content, S2 derived fibroblasts showed a more severe OxPhos deficient phenotype than S1 cells. The levels of mtDNA-derived transcripts were lower than those in S1, and several subunits of the respiratory chain complexes were absent. Thus, mtDNA levels did not correlate with mitochondrial RNA levels as expected (Gomez-Duran et al., 2012) indicating that FBXL4 deficiency also affects the correct mtDNA expression.

The patient primary fibroblasts showed augmented mitochondrial mass, when assessed by MitoTracker Green fluorescent staining. The increase was particularly high in S2 cells. It can be speculated that FBXL4 deficient fibroblasts might have overcome the defect in mtDNA maintenance by expanding their mitochondrial mass as an adaptation to the life in culture. An attempt to compensate for OxPhos defects increasing mitochondrial mass resembles the massive mitochondrial proliferation observed in muscle of patients with mtDNA-related diseases (resulting in ragged-red fibers) (DiMauro and Schon, 2003). However, in muscle from FBXL4 deficient patients no ragged red fibers have been observed (Gai et al., 2013; Morton et al., 2017), although they are a common feature in newborns and infants with mitochondrial disorders (Jou et al., 2019).

Previous patient reports have described a hyperfragmentation of the mitochondrial network in affected fibroblasts (Bonnen et al., 2013; Gai et al., 2013; Antoun et al., 2016). This has lead to classify FBXL4 as protein participating in mitochondrial dynamics (El-Hattab et al., 2017a). The highly compacted mtDNA–protein complexes or nucleoids, that usually can be visualized as punctate structures evenly distributed within the mitochondrial network, have also been found altered, enlarged, and clustered (Bonnen et al., 2013). Our patients' fibroblasts also presented a mitochondrial network fragmented into multiple small mitochondria. Interestingly, S1 fibroblasts partially recovered a connected mitochondrial network when functionally complemented with wt-*FBXL4*, whereas mitochondria in S2 complemented fibroblasts remained mostly punctuate. These findings suggest that mitochondrial fragmentation could be secondary to the metabolic defects induced by absence of the FBXL4 protein. Augmented mitochondrial division could be related with attempts to increase mtDNA synthesis because both processes are closely related. In mammalians, tubular ER-mitochondria contacts, by an unknown mechanism, connect the sites of mitochondrial division with the subset of nucleoids engaged in mtDNA synthesis. Thus, following division, nucleoids segregate to both tips of daughter mitochondria (Lewis et al., 2016). This fact, however, could not be confirmed in our studies.

Increased mitochondrial division can also precede mitophagy, because it divides elongated mitochondria into pieces that can be engulfed by autophagosomes to regulate their number and maintain quality control (Youle and Narendra, 2011). Both S1 and S2 fibroblasts demonstrated increase of the autophagic-vesicles coating-protein LC3B-II, suggesting that FBXL4 deficiency promotes autophagic processes. In genetically complemented S1 cells autophagosome formation decreased. It could be speculated that it reflects the need for selective elimination of the organelles lacking a functional OxPhos system and/or the necessity of recycling intracellular components to compensate for the starvation-like situation (Geng and Klionsky, 2008). The reduction of mitochondrial components other than subunits of the respiratory chain clearly observed in S2 can be reflecting its elimination by mitophagy. In genetically complemented S2 cells, the increased autophagy persisted possibly regulating the organelle number since the abnormally high increase in mitochondrial membrane also persisted.

In contrast to patients with typical MDSs due to a disorder of mtDNA replication or nucleoside salvage/synthesis, in patients with FBXL4 deficiency the mtDNA/CS ratio is normal (Huemer et al., 2015). Our patient cells also presented CS deficiency. It can be a secondary effect, because interruptions to the respiratory chain can affect the TCA cycle flux (Vafai and Mootha, 2012). Deficiency in another TCA cycle enzyme, succinyl-CoA synthetase (SCS), has also been associated with an encephalomyopathic form of MDS. However, the involvement of SCS in MDS seems to be related to a role of the enzyme in the mitochondrial nucleoside salvage pathway, facilitating the conversion of dNDPs to dNTPs (Viscomi and Zeviani, 2017).

In summary, this work provides evidence of the pathogenicity of novel variants in FBXL4, demonstrating that FBXL4 is necessary not only for the homeostasis but also for the expression of mtDNA. Since in S2 fibroblasts the rescue of the bioenergetics defects by the *FBXL4* gene can occur independently of the recovery of control mitochondrial mass, mitochondrial network, or autophagic levels, these could be compensatory and subsequent to the bioenergetic defects. Most F-box proteins function as adaptors in phosphorylation dependent ubiquitination-complexes (Craig and Tyers, 1999). A role in post-translational modification of the mitochondrial proteome could reconciled the disparity of effects observed in defective FBXL4, but further work is required to determine the precise molecular function.

## Data Availability Statement

The datasets Generated for this study can be found in NCBI https://www.ncbi.nlm.nih.gov/bioproject/PRJNA590845 and https://www.ncbi.nlm.nih.gov/bioproject/PRJNA592374.

## Ethics Statement

The studies involving human participants were reviewed and approved by ethics committee of the Government of Aragón (CEICA CP- 12/2014). Written informed consent to participate in this study was provided by the participants' legal guardian/next of kin.

## Author Contributions

MO, JO-E, AG-C, MI, and MG-C were responsible for the sample collection, analysis of clinical data, and treatment. RA, DY, and AF-M participated in the biochemical and molecular genetic diagnostic studies of the patients. SE, NG-P, JA-G, PG, and JA-S participated in the biochemical and molecular analysis of patient samples. ER-P, JM, and MB-B were responsible of study design, data interpretation, and drafting of the manuscript. All authors have critically reviewed and they approved the final manuscript.

## Funding

This work was supported by grants from Instituto de Salud Carlos III (PI17/00021, PI17/00166, and PI/1700109); Fundación Mutua Madrileña; Precipita-FECYT Crowdfunding program (PR194); Gobierno de Aragón (Grupos Consolidados B33_17R); FEDER 2014-2020 “Construyendo Europa desde Aragón” and Asociación de Enfermos de Patología Mitocondrial (AEPMI). The CIBERER is an initiative of the ISCIII.

## Conflict of Interest

The authors declare that the research was conducted in the absence of any commercial or financial relationships that could be construed as a potential conflict of interest.

The handling editor declared a past collaboration with two of the authors SE, JM.

## References

[B1] Andreu A. L. Martinez R. Marti R. Garcia-Arumi E. (2009). Quantification of mitochondrial DNA copy number: pre-analytical factors. Mitochondrion 9 (4), 242–246. 10.1016/j.mito.2009.02.006 19272467

[B2] Antoun G. McBride S. Vanstone J. R. Naas T. Michaud J. Redpath S. (2016). Detailed biochemical and bioenergetic characterization of FBXL4-related encephalomyopathic mitochondrial DNA depletion. JIMD Rep. 27, 1–9. 10.1007/8904_2015_491 26404457 PMC5580732

[B3] Bonnen P. E. Yarham J. W. Besse A. Wu P. Faqeih E. A. Al-Asmari A. M. (2013). Mutations in FBXL4 cause mitochondrial encephalopathy and a disorder of mitochondrial DNA maintenance. Am. J. Hum. Genet. 93 (3), 471–481. 10.1016/j.ajhg.2013.07.017 23993193 PMC3769921

[B4] Craig K. L. Tyers M. (1999). The F-box: a new motif for ubiquitin dependent proteolysis in cell cycle regulation and signal transduction. Prog. Biophys. Mol. Biol. 72 (3), 299–328. 10581972 10.1016/s0079-6107(99)00010-3

[B5] DiMauro S. Schon E. A. (2003). Mitochondrial respiratory-chain diseases. N Engl. J. Med. 348 (26), 2656–2668. 10.1056/NEJMra022567 12826641

[B6] El-Hattab A. W. Craigen W. J. Scaglia F. (2017a). Mitochondrial DNA maintenance defects. Biochim. Biophys. Acta Mol. Basis Dis. 1863 (6), 1539–1555. 10.1016/j.bbadis.2017.02.017 28215579

[B7] El-Hattab A. W. Dai H. Almannai M. Wang J. Faqeih E. A. Al Asmari A. (2017b). Molecular and clinical spectra of FBXL4 deficiency. Hum. Mutat. 38 (12), 1649–1659. 10.1002/humu.23341 28940506

[B8] Emperador S. Pacheu-Grau D. Bayona-Bafaluy M. P. Garrido-Perez N. Martin-Navarro A. Lopez-Perez M. J. (2014). An MRPS12 mutation modifies aminoglycoside sensitivity caused by 12S rRNA mutations. Front. Genet. 5, 469. 10.3389/fgene.2014.00469 25642242 PMC4294204

[B9] Fernandez-Marmiesse A. Perez-Poyato M. S. Fontalba A. Marco de Lucas E. Martinez M. T. Cabero Perez M. J. (2019). Septo-optic dysplasia caused by a novel FLNA splice site mutation: a case report. BMC Med. Genet. 20 (1), 112. 10.1186/s12881-019-0844-5 31234783 PMC6591933

[B10] Gai X. Ghezzi D. Johnson M. A. Biagosch C. A. Shamseldin H. E. Haack T. B. (2013). Mutations in FBXL4, encoding a mitochondrial protein, cause early-onset mitochondrial encephalomyopathy. Am. J. Hum. Genet. 93 (3), 482–495. 10.1016/j.ajhg.2013.07.016 23993194 PMC3769923

[B11] Geng J. Klionsky D. J. (2008). The Atg8 and Atg12 ubiquitin-like conjugation systems in macroautophagy. ‘Protein modifications: beyond usual suspects' review series. EMBO Rep. 9 (9), 859–864. 10.1038/embor.2008.163 18704115 PMC2529362

[B12] Gomez-Duran A. Pacheu-Grau D. Martinez-Romero I. Lopez-Gallardo E. Lopez-Perez M. J. Montoya J. (2012). Oxidative phosphorylation differences between mitochondrial DNA haplogroups modify the risk of Leber's hereditary optic neuropathy. Biochim. Biophys. Acta 1822 (8), 1216–1222. 10.1016/j.bbadis.2012.04.014 22561905

[B13] Huemer M. Karall D. Schossig A. Abdenur J. E. Al Jasmi F. Biagosch C. (2015). Clinical, morphological, biochemical, imaging and outcome parameters in 21 individuals with mitochondrial maintenance defect related to FBXL4 mutations. J. Inherit Metab. Dis. 38 (5), 905–914. 10.1007/s10545-015-9836-6 25868664 PMC4841446

[B14] Jou C. Ortigoza-Escobar J. D. O'Callaghan M. M. Nascimento A. Darling A. Pias-Peleteiro L. (2019). Muscle involvement in a large cohort of pediatric patients with genetic diagnosis of mitochondrial disease. J. Clin. Med. 8 (1). 10.3390/jcm8010068 PMC6352184 30634555

[B15] Kirby D. M. Thorburn D. R. Turnbull D. M. Taylor R. W. (2007). Biochemical assays of respiratory chain complex activity. Methods Cell Biol. 80, 93–119. 10.1016/S0091-679X(06)80004-X 17445690

[B16] Klionsky D. J. Abeliovich H. Agostinis P. Agrawal D. K. Aliev G. Askew D. S. (2008). Guidelines for the use and interpretation of assays for monitoring autophagy in higher eukaryotes. Autophagy 4 (2), 151–175. 10.4161/auto.5338 18188003 PMC2654259

[B17] Lewis S. C. Uchiyama L. F. Nunnari J. (2016). ER-mitochondria contacts couple mtDNA synthesis with mitochondrial division in human cells. Science 353 (6296), aaf5549. 10.1126/science.aaf5549 27418514 PMC5554545

[B18] Morton S. U. Neilan E. G. Peake R. W. A. Shi J. Schmitz-Abe K. Towne M. (2017). Hyperammonemia as a presenting feature in two siblings with FBXL4 Variants. JIMD Rep. 35, 7–15. 10.1007/8904_2016_17 27858371 PMC5585110

[B19] Perales-Clemente E. Bayona-Bafaluy M. P. Perez-Martos A. Barrientos A. Fernandez-Silva P. Enriquez J. A. (2008). Restoration of electron transport without proton pumping in mammalian mitochondria. Proc. Natl. Acad. Sci. U.S.A. 105 (48), 18735–18739. 10.1073/pnas.0810518105 19020091 PMC2585044

[B20] Pronicka E. Piekutowska-Abramczuk D. Ciara E. Trubicka J. Rokicki D. Karkucinska-Wieckowska A. (2016). New perspective in diagnostics of mitochondrial disorders: two years' experience with whole-exome sequencing at a national paediatric centre. J. Transl. Med. 14 (1), 174. 10.1186/s12967-016-0930-9 27290639 PMC4903158

[B21] Suomalainen A. Isohanni P. (2010). Mitochondrial DNA depletion syndromes–many genes, common mechanisms. Neuromuscul Disord. 20 (7), 429–437. 10.1016/j.nmd.2010.03.017 20444604

[B22] Vafai S. B. Mootha V. K. (2012). Mitochondrial disorders as windows into an ancient organelle. Nature 491 (7424), 374–383. 10.1038/nature11707 23151580

[B23] Viscomi C. Zeviani M. (2017). MtDNA-maintenance defects: syndromes and genes. J. Inherit Metab. Dis. 40 (4), 587–599. 10.1007/s10545-017-0027-5 28324239 PMC5500664

[B24] Youle R. J. Narendra D. P. (2011). Mechanisms of mitophagy. Nat. Rev. Mol. Cell Biol. 12 (1), 9–14. 10.1038/nrm3028 21179058 PMC4780047

[B25] Yubero D. Montero R. Martin M. A. Montoya J. Ribes A. Grazina M. (2016). Secondary coenzyme Q10 deficiencies in oxidative phosphorylation (OXPHOS) and non-OXPHOS disorders. Mitochondrion 30, 51–58. 10.1016/j.mito.2016.06.007 27374853

